# Supplementary material for: Accurate prediction of flux distributions compatible with metabolite concentration effects in genome-scale metabolic networks
Source: PLoS Comput Biol. 2026 Mar 16;22(3):e1014066. doi: 10.1371/journal.pcbi.1014066 (PMC13008253; doi:10.1371/journal.pcbi.1014066)
Supplement: Attachment — Submitted filename: PLoS_reviews.docx [file pcbi.1014066.s006.docx]

**Response Letter to “Accurate prediction of flux distributions compatible with metabolite concentration effects in genome-scale metabolic networks” (PCOMPBIOL-D-25-01676)**

In the following, we provide a point-by-point response to all reviewers’ comments. For completeness, we reproduce the comments in a regular font; our responses are provided in a bold font.

**Reviewers' comments:**

Reviewer #1: Soleymani et al. introduce KineFlux, a flux balance analysis (FBA) approach that integrates machine learning with enzyme-constrained modeling to incorporate metabolite effects. While the methodology appears elegant, its novelty requires more thorough demonstration. I hope the following comments will help improve the manuscript.

First, I do not believe the primary novelty of KineFlux lies in its ability to predict more accurate fluxes compared to other FBA methods. If the authors intend to claim this as a key advance, they should provide supporting evidence e.g. by demonstrating that KineFlux outperforms state-of-the-art FBA methods in flux predictions.

Furthermore, it remains unclear whether there exists any specific scenario where only KineFlux is applicable, while other methods are not. The authors note that KineFlux can predict flux distributions using only quantitative proteomics data (Lines 15-16). However, classical FBA can also predict fluxes with only a few exchange rates as constraints, which are often more experimentally accessible than omics data. Even enzyme-constrained FBA models can predict fluxes without any experimental input by simply maximizing the growth rate while allowing unlimited nutrient uptake.

**We would like to thank the reviewer for the constructive criticism. To address the concern about the prediction of model accurate fluxes resulting from KineFlux, we compared the fluxes predicted by KineFlux with those obtained from FBA, pFBA, and the enzyme-constrained GEM of *E. coli* iJO1366. The estimated fluxes derived from ^13^C Metabolic Flux Analysis (^13^C-MFA) were used as the ground truth. Based on the evaluation metrics (Pearson correlation coefficients and mean squared error), our approach significantly outperforms the aforementioned constraint-based methods, both in condition-specific flux distributions and in per-reaction fluxes across conditions. The corresponding results are presented in the second section of the results and Table 1.**

**Clearly, the applicability of constraint-based approaches is determined by the requirements of the approaches. Since all constraint-based approaches predict fluxes (in addition to other metabolic / physiological traits), we see no specific condition under which KineFlux can be exclusively applied. Similar to other constraint-based approaches, KineFlux requires information on the growth conditions; however, KineFlux specifically requires quantitative enzyme abundance data for flux prediction. Given the challenges in gathering labeling data (especially from instationary labeling experiments, required to study nitrogen metabolism in any organism), KineFlux offers a valuable addition that relies on quantitative proteomics data that are becoming feasible to gather at a genome-wide scale. We have expanded the Discussion section in the updated version of the manuscript.**

In my view, the true novelty of KineFlux may lie in its ability to infer reactions regulated by metabolite concentrations. This aspect should be emphasized and further demonstrated in a revised version. The authors could focus more on the predicted metabolite-mediated regulation e.g. by evaluating how many of these predictions are supported by existing experimental evidence from literature and databases, and explaining why KineFlux might fail to capture some known regulatory effects. Presenting these results in the main figures, rather than only discussing them in the text, would strengthen the argument.

**We appreciate this comment. To address this point, we have added a new subsection evaluating the predicted enzyme regulators. Specifically, we assessed the identified regulators using the BRENDA database as well as evaluating the STITCH database. While only a few matches were found between our predicted regulators and those reported in BRENDA, a more comprehensive assessment was possible using STITCH, which contains a broader set of interaction data. As our approach was not primarily designed for regulator prediction, we did not expect it to outperform dedicated feature-based machine learning or deep learning methods (Habibpour *et al.*, 2024, Metabolic Engineering; Campana *et al.*, 2023, NAR Genomics & Bioinformatics). The corresponding results could be found in third section of the results.**

Although the authors mention that KineFlux utilizes enzyme-constrained models, they do not detail how the framework integrates such constraints. The Methods section lacks information on the construction of enzyme-constrained models for E. coli and S. cerevisiae. Additionally, it would be valuable to clarify whether KineFlux can be applied to classical metabolic models without enzyme constraints.

**Based on our previous observations, the first term of the objective function typically approaches zero due to the small value of w used. Considering the relationship v=E·k_app_·𝜂, where 𝜂 is in the range [0,1], it follows that v<=E⋅k_app_. To ensure that enzyme capacity constraints were properly enforced, we explicitly added the corresponding constraints as defined in Eq (7) and re-ran the pipeline and updated the findings. This modification did not significantly affect the results.**

In addition, I have some minor comments below:

Lines 24-25: “without knowledge of enzyme kinetics”, but the enzyme-constrained models already include enzyme kinetics.

**The enzyme-constrained models do not include information about enzyme kinetics (i.e., the effect of metabolite concentration on fluxes). We have better specified this in the updated version of the manuscript.**

Fig 1D: what is the meaning of the adjusted R2 value? Does it correspond to the coefficient for the fitting curve in Fig 1E?

**The adjusted coefficient of determination is a modified form of the standard R^2^ metric that accounts for the number of predictors included in the model.**

Line 94: double “that”.

**Thanks for pinpointing it, we corrected it.**

Line 235: the authors mentioned Warburg effect but the reference [18] is about S. cerevisiae. Maybe the authors meant Crabtree effect?

**This is correct and was modified in the updated version of the manuscript.**

Reviewer #2: The manuscript presents a method for predicting the modulation of metabolic fluxed by non-substrate metabolites. For this, the authors introduce a function ‘eta’ that takes values between zero and one and gets multiplied with a predicted flux to account for regulatory effects of other metabolites. The function eta is modelled as a logistic regression estimated from metabolomic and proteomic datasets, with at most three non-substrate metabolites in used as covariates. Overall, the method looks promising and the authors demonstrate the applicability on a number of datasets from E. coli and yeast.

Nevertheless, the manuscript has a number of major shortcomings that I would like to see addressed:

1) Please be clearer and more precise when you use which data. In particular, some datasets are 13C measurements while others are pFBA predictions and it is sometimes unclear which you use. I am also unclear why these two data should have the same status, as pFBA ‘only’ provides predictions, while 13C are actual measurements.

**We appreciate the comment. Our primary goal was to use experimentally estimated fluxes paired with quantitative proteomics data as input for our pipeline. However, such data were only available for the *E. coli* from the study of Heckmann *et al.*, which is based on flux bounds derived from ^13^C MFA and therefore served as the main focus of our manuscript. For the datasets from Davidi *et al.* and for yeast, where fluxomic measurements were not available, we used pFBA-derived fluxes as an alternative. This is justified given that the studies for estimation of proxies for enzyme turnover numbers in the studies from Milo’s and Nielsen’s labs (Davidi *et al.*, 2016, PNAS; Chen *et al.*, 2021, PNAS) made use of the same approaches.**

2) It was unclear to me how exactly the covariates for the logistic regression were chosen. E.g., with 100 possible metabolites you would already have ~160.000 models with exactly three covariates to choose from.

**We would like to thank the reviewer for raising this concern. We filtered out metabolites that exhibited zero flux-sum values across all conditions, and additionally applied a threshold on the coefficient of variation to remove low-variability metabolites. As a result, the total number of metabolite–reaction combinations was fewer than 200,000 in the worst case. To manage the computational cost, we parallelized the pipeline to efficiently process this number of combinations.**

3) For the covariates chosen in the end: how clear-cut was this decision. Are there other sets of metabolites that would provide similar fit and predictive accuracy? Did you check which metabolites your method chose and if they make biological sense as regulators for a specific enzymatic reaction?

**We appreciate this comment. To address this point, we have added a new subsection evaluating the predicted enzyme regulators. Specifically, we assessed the identified regulators using the BRENDA database as well as evaluating the STITCH database. While only a few matches were found between our predicted regulators and those reported in BRENDA, a more comprehensive assessment was possible using STITCH, which contains a broader set of interaction data. As our approach was not primarily designed for regulator prediction, we did not expect it to outperform dedicated feature-based machine learning or deep learning methods. The corresponding results could be found in third section of the results**.

4) How exactly did you perform the cross-validation? Your dataset contains duplicates of the same conditions, so arbitrary splitting will likely produce training and test sets that contain one duplicate each of the same condition. This would not provide a valid error estimate.

**Thank you for your insightful comment. To address this point, we applied Group K-Fold cross-validation, ensuring that biological replicates were assigned together to either the training or test set, thereby preventing any data leakage. We subsequently re-ran the entire pipeline and updated the results accordingly. The corresponding updates are included in second section of the results, lines 121-124**.

5) I did not understand the reasoning for your subsystem analysis. What can we learn from this regarding the accuracy of your method?

**Thank you for your comment. This analysis was added to further investigate how the proposed method predicts fluxes across different subsystems, identifying which subsystems exhibit better predictive performance and which show lower accuracy.**

6) For the subsystems: You use a zero-one criterion effectively counting how many values fall within a prediction interval. But that would mean that a very bad model with very wide prediction interval will perform excellently according to your criterion.

**Thank you for your comment. We have added Mean Squared Error (MSE) to the evaluation metrics and compared the predicted fluxes with those obtained from other constraint-based approaches, using the ^13^C MFA flux estimations as the ground truth. The results indicate that the high proportion of reactions falling within the prediction interval reflects the strong predictive performance of KineFlux.**

7) Similarly, why do you choose the correlation coefficient as a measure of predictive power? And why do you choose different cut-offs for a ‘good’ coefficient (0.8 sometimes, other times 0.6).

**Thank you for your comment. We added the Mean Squared Error (MSE) as an additional evaluation metric. There is a trade-off in selecting the threshold value: increasing the threshold reduces the number of reaction-specific regressor functions that can be integrated into the optimization problem, causing the predicted flux distribution to converge toward the pFBA solution due to the second term in the objective function. Conversely, lowering the threshold increases the number of reactions included in the optimization but may introduce regressors with low predictive accuracy for η, making it more difficult to obtain an optimal solution.**

8) With your various analyses I still remain somewhat unconvinced of the power of your method. One good comparison would be of the flux prediction with and without the ‘eta-correction’ from your method. How much do fluxes actually change when accounting for the metabolic flux effects?

**We would like to thank the reviewer for the constructive criticism. To address the concern about the prediction of model accurate fluxes resulting from KineFlux, we compared the fluxes predicted by KineFlux with those obtained from FBA, pFBA, and the enzyme-constrained GEM of *E. coli* iJO1366. The estimated fluxes derived from ^13^C Metabolic Flux Analysis (^13^C-MFA) were used as the ground truth. Based on the evaluation metrics (Pearson correlation coefficients and mean squared error), our approach significantly outperforms the aforementioned constraint-based methods, both in condition-specific flux distributions and in per-reaction fluxes across conditions. The corresponding results are presented in second section of the results and Table 1.**

9) Please provide some information about the distribution of ‘eta’ over conditions and reactions/enzymes. Does this function actually contribute to modulating the flux? Or are the values close to one anyway?

**To address this concern, we analyzed the distribution of η values from both reaction-based and condition-based perspectives. The results, presented in second section of the results** **, show that the η values are not concentrated near one but instead hover around 0.5 with relatively high standard deviations, facilitating the application of machine learning approaches.**

10) Your two studies use KO data for E. coli and growth conditions for yeast. These are biologically very different conditions. What is the reasoning that these are treated as equivalent?

**We did not intend to suggest that gene knockouts and growth conditions are equivalent. Rather, our goal was to demonstrate the broader applicability of our approach across different experimental scenarios – a must for any machine-learning-based approach, often tending to suffer from lack of transferability between experimental scenarios.**

Minor:

1) There is no mention of alternative methods, but much has been published on integrating and using different -omics data for metabolic models. Please provide some context how your method fits into this landscape.

**We have provided references to reviews dedicated to integration of enzyme abundances and metabolite concentrations into constraint-based models. We expanded the section of the introduction to include an explicit mention of model balancing as an alternative to SIMMER; we are not aware of other approaches where similar research directions have been followed.**

2) How is your method related to ‘model balancing’ (if at all)? This seems to address a very similar problem.

**Model balancing was developed to estimate enzyme turnover numbers for all enzyme functions in a system at once (in contrast to approaches like SIMMER). In addition, model balancing relies on modular rate laws, described by a Michaels-Menten like form; note that KineFlux does not rely on such a form of enzyme kinetics, and instead builds machine learning models (here, logistic regression for the purpose of their usage as constraints; note that other machine learning models that can be explicitly written in a mathematical form may also be considered).**

3) You discuss ‘metabolic concentration effects’ throughout the manuscript and use the term in the title. But a somewhat vague definition only occurs in the method section after page 10. Please provide some definition or at least description of what you mean early on, as this concept is central to your method.

**We have provided a description in the introduction, and point out that precise definition appeared already in the first section of the results.**

4) What were the growth conditions in your dataset? Are they comparable or do they include different carbon sources, for example?

**Yes, the dataset includes multiple carbon sources for growth. In the Heckmann dataset, the knockout strains were under various carbon sources, including glucose, acetate, succinate, and lactate.**

5) Your method hinges a lot on the maximal apparent k_cat, which you estimate as the maximal observed value in the data. Why is this a useful estimate? Could one improve the method by considering the distribution of this maximum?

**Thank you for your comment. We investigated the effect of k_app,max_ in the sixth section of the results**. **The k_app,max_ values derived from gene knockout strains resulted in better flux predictions, likely because these knockouts induce strong physiological perturbations and associated high growth rates, leading to more robust *in vivo* kcat estimates.**

Reviewer #3: In this work, the authors develop a hybrid machine learning and constraint-based metabolic modeling method termed KineFlux to integrate enzyme cost considerations with metabolic concentration effects. They attempt to model the k_apparent parameter, which is an outcome of metabolite concentration effects, by obtaining a relationship between this parameter and a metabolic concentration proxy obtained by computed flux-sums. The authors then use the inferred relationship to design a constraint-based metabolic modeling approach to incorporate the modeled metabolite concentration effects, thereby (hopefully) obtaining a more accurate flux simulation. It’s an interesting and clever method that seems to have conceptual merit.

The practical question is given the series of sequential approximations necessary to implement the approach, how well does it work in the end? My initial questions were 1) whether there is sufficient proteomics data to accurately train the estimator model, 2) whether flux-sums are sufficiently accurate proxies for metabolite concentrations, and 3) whether pFBA is a sufficient method for accurately estimating flux (especially given that the point of the developed method is seemingly to improve on pFBA predictions). The answers to these questions primarily depend on the final performance of the method, as it is difficult to determine their answers independently given lack of data on each component.

My concerns with the work as presented are primarily 1) a lack of controls to which the method’s performance can be compared (especially given that pFBA is often quite good at estimating fluxes), and 2) a lack of detailed investigation of the metabolite effects, in terms of presenting overall characteristics of the estimated metabolite effects as well as specific case studies that could build confidence in the method’s predictions.

I hope that the authors can expand on their work to build confidence that the predictions made by the method are meaningful. My specific comments are below.

Major Comments

- Primary issue 1: Lack of control/reference comparisons. The authors produce a variety of parity plots throughout the manuscript comparing their model’s predictions to ‘data’ (pFBA flux simulations?), which they claim is evidence of improved predictions. However, in Figure 2 for example, it is not clear to me if the authors are making a completely independent prediction or just recovering the relationships the model was trained on. It would be good to compare this to a control, such as logit models trained on permuted data for example, as well as examining performance using holdout data. It also seems like these predictions should be compared to uncorrected pFBA-based flux predictions, and predictions with random etas assigned within an expected range, for example.

**We would like to thank the reviewer for the constructive criticism. To address the concern about the prediction of model accurate fluxes resulting from KineFlux, we compared the fluxes predicted by KineFlux with those obtained from FBA, pFBA, and the enzyme-constrained GEM of *E. coli* iJO1366. The estimated fluxes derived from ^13^C Metabolic Flux Analysis (^13^C-MFA) were used as the ground truth. Based on the evaluation metrics (Pearson correlation coefficients and mean squared error), our approach significantly outperforms the aforementioned constraint-based methods, both in condition-specific flux distributions and in per-reaction fluxes across conditions. The corresponding results are presented in second section of the resultsand Table 1.**

**To make sure there is no data leakage in the pipeline we applied Group K-Fold cross-validation, ensuring that biological replicates were assigned together to either the training or test set. Furthermore, KineFlux was evaluated under entirely unseen conditions, with the corresponding results reported in page 6, lines 121-124.**

- Primary issue 2: Lack of details on results. The authors present a variety of parity plots but very little detailed discussion of 1) what the intermediate steps look like (for example the distribution of metabolite effects (eta), or some description of what the logit models look like on a per-reaction basis – how much do they change? What range of values is expected?) and 2) specific reactions exhibiting significant metabolite concentration effects, such as the MDH as discussed by Milo and colleagues in their original paper describing k_apparent_max (PMID: 26951675).

**To address this concern, we analyzed the distribution of η values from both reaction-based and condition-based perspectives in the training data and in the optimization solutions. The results, presented in second section of the results, show that the η values are not concentrated near one but instead hover around 0.5 with relatively high standard deviations, facilitating the application of machine learning approaches. The η value for the reaction MDH was 0.24 ± 0.19, indicating a significant effect on turnover number, consistent with the findings reported in the referenced study.**

- Issue 3: It is not clear to me which “v_data” values are from 13C MFA vs pFBA. In line 208, the authors state “Second, the discrepancy may arise from the differing sources of flux estimates: the flux estimates in the unseen condition are based on pFBA, whereas the training data relies on more accurate 13C MFA.” In Figure 5, which is the unseen conditions example, the axes still say v_data and not v_pFBA or similar. The axes should be clearly labeled throughout with the source of the flux values, whether C13 MFA or pFBA.

**We changed the axis titles for Fig 4 B and D and also Fig 5 B.**

- Issue 4: I am concerned about the subsystem analysis in Figure 3B. It seems that the model claims to predict fluxes well in peripheral metabolism but performs notably more poorly in central metabolism (glycolysis, pentose phosphate pathway, TCA cycle). Typically peripheral metabolism is tightly constrained by growth rate in these models while central metabolism has more flexibility. I can’t help but wonder if these statistics are inflated by hard constraints in the model. Can the authors please add appropriate control comparisons, such as the uncorrected pFBA-based flux predictions, and predictions with random etas assigned within an expected range, for example?

**Thank you for your comment. We have added a comparison between the predicted fluxes from KineFlux and other established approaches *e.g.* pFBA and FBA.**

Minor Comments

- Line 49 “the flux predictions resulting from enzyme-constrained models are obtained under the implicit assumption of saturation of enzymes by their substrates” – this is not true (I can’t speak for all methods, but many do not make this assumption). A keff is assumed at some level of saturation that is assumed condition invariant, but not necessarily saturated.

**We would like to thank the reviewer for pointing this out; we agree that some level of saturation is assumed – that is the same across conditions and enzymes (included in the Ptot constraint).**

- Line 63 – No need for flowery but empty adjectives like an ‘innovative combination’.

**We have removed the “innovative”**

- Line 83: “Fluxomic data can be obtained by applying parsimonious flux balance analysis (pFBA)” – pFBA does not provide “fluxomic data” but rather “fluxomic predictions”

**We incorporated flux bounds derived from the confidence intervals obtained through ^13^C MFA experiments. Since these data were not available for all reactions, the remaining fluxes were estimated using parsimonious flux balance analysis (pFBA) using the ^13^C MFA bounds.**

- Line 93 and the corresponding paragraph – “flux-sums” (defined in the caption of Figure 1 as the sum of fluxes producing a metabolite) are not adequately introduced for the reader to understand this section. A longer discussion of the previous work here and the principles underlying the connection between flux-sums and metabolite concentrations would be warranted here to justify the approach.

**We referred to the study by Seyis *et al.* (2025, PLOS Computational Biology) which investigated this aspect and utilized flux-sum values as proxies for metabolite concentrations.**

- For many of the scatter plots throughout the paper (e.g. Figure 5B), adding labels for at least a readable portion of non-trivial off diagonal elements would be a big improvement, so that the reader can better understand where the model has inaccuracies.

**We have added labels for the reactions that have non-zero values for the x- and y-axis variables and are located farthest from the diagonal in the Figures 3A, 4D, and 5B.**
