# Supplementary material for: Accurate prediction of flux distributions compatible with metabolite concentration effects in genome-scale metabolic networks
Source: PLoS Comput Biol. 2026 Mar 16;22(3):e1014066. doi: 10.1371/journal.pcbi.1014066 (PMC13008253; doi:10.1371/journal.pcbi.1014066)
Supplement: Attachment — Submitted filename: ResponseToReviewers_v3.docx [file pcbi.1014066.s007.docx]

**Response to the reviewers’ comments to**

**“Accurate prediction of flux distributions compatible with metabolite concentration effects in genome-scale metabolic networks”**

The response letter includes the point-by-point responses to the reviewers’ comments. For completeness, the reviewers’ comments are replicated in a regular font, and our replies appear in a bold font.

Reviewer #1: While the authors responded well to most of my previous comments, I do not think that they adequately addressed the following comment:
“Although the authors mention that KineFlux utilizes enzyme-constrained models, they do not detail how the framework integrates such constraints. The Methods section lacks
information on the construction of enzyme-constrained models for E. coli and S.
cerevisiae. Additionally, it would be valuable to clarify whether KineFlux can be applied
to classical metabolic models without enzyme constraints.” This comment requested details on model construction and discussion on applicability of KineFlux to classical metabolic models. However, the authors provided an irrelevant response.

**The enzyme constraints are considered only for unienzyme catalysed reactions by using the constraint typical for enzyme-constrained models, i.e., v<=k_cat_*E, in Eq (7). In addition, we can include a total protein constraint, in the updated Eq. (7), which we omitted from the earlier version. The reason is that E is not a variable, but is fixed by the data. As a result, KineFlux requires the use of enzyme abundances and information about enzyme catalytic rates. This also clarifies that KineFlux requires ecGEMs. We hope this answer resolves the issue.**

Reviewer #2: I thank the authors for the improvements in their manuscript. In particular the use of data sets, the cross-validation procedures, and the explanation of the method as such are now much clearer. I also appreciate the changes to the Introduction, which now more clearly states the aims and central ideas of the manuscript.

**We would like to thank the reviewer for acknowledging the improvements in the revised version of the manuscript. The remaining points are addressed in our answers below.**

Despite the many improvements, I believe that several major issues have not yet been addressed appropriately in this revision.

1) What exactly is the proposed use-case for this method? You argue that the method consistently gives predictions close to the pFBA predictions. So why to use the established and trusted pFBA/FBA in the first place? What cases does the proposed method cover that the current methods do not?

**Already in the last revision we have thoroughly compared the predicted fluxes with other constraint-based approached included pFBA, for which the results are shown in Table 1 in the manuscript. Our findings demonstrate that the predicted fluxes by KineFlux significantly outperform those obtained by pFBA when flux estimates from 13C labeling studies are used as a reference (which is well-established in fluxomics analysis). For instance, the MSE of log-transformed flux distributions for KineFlux is 4.464±1.530, while for pFBA it is at least two-fold higher, at 9.372±1.539. In addition, fluxes of individual reactions have MSE of 0.388±1.744 and 0.677±3.213 for KineFlux and pFBA, respectively, demonstrating the improved performance that is quantified by additional measures (see Table 1).**

2) Similar to the other reviewers, I believe the method is interesting and has merit, but that the claim that it captures metabolite concentration effects has not been sufficiently demonstrated. If true, then the method would add a very valuable perspective on regulation in genome-scale metabolic networks. However, your comparisons with BRENDA and STITCH seem to indicate that the method almost never picks known regulatory metabolites for the logistic regression model. Could you expand on the use of your method for identifying key regulators of reactions and/or reactions that are highly regulated (in specific conditions)? As a minor point: why do you use additional metabolites _not_ identified as regulators in your logistic regression for comparison to the databases?

**The flux of a reaction is determined by the concentration of active enzymes as well as concentration of metabolites that either act as substrates or as effectors (activators / inhibitors) of fluxes. If we were to only include the effect of substrates, we would neglect the important effect that metabolite regulation has on fluxes. This explains the reasons why we include metabolites other than substrates in the logistic regression.**

**To the comparison with BRENDA and STITCH, we recognize that the logistic regression models are non-unique, in the sense that different combination of substrates and additional regulators may provide as good predictive power as the models we have selected for use. Therefore, the performance of our approach against BRENDA and STITCH provides a lower bound on the accuracy of the retrieved regulators. For instance, considering metabolites highly correlated with the regulators included in the model can also be used as a strategy to evaluate the retrieval rate by KineFlux. This is what we inspected by considering metabolites whose flux sums are correlated with a value of at least 0.8 to the flux sums of metabolites present in the logistic models. In the previous version, we reported only the precision, and now include the recall rate which is 4.39% for BRENDA database and 8.13% for STITCH database. We added a comment to this limitation in the discussion of the updated manuscript.**

**Since the flux predictions from KineFlux improve over three contending approaches (FBA and pFBA with GEM, FBA with ecGEM), the interpretability of the models can be further improved by not considering the penalization of the logistic regression with the number of predictors (note that we consider the adjusted coefficient of determination to have a statistically sound model selection). The reason is that statistical soundness may be confounding biological reasoning.**

3a) I maintain my previously comment that both the Pearson correlation coefficient is inadequate for evaluation comparing KineFlux and (p)FBA prediction, at least as reported. Clearly, you can get a correlation of one with _any_ linear relationship between estimate and prediction, which says nothing about the accuracy of the prediction. For example, Fig 2B clearly shows consistent underestimation of fluxes by KineFlux yet yields an excellent correlation.

**We would like to thank the reviewer for raising this important point. It is for this reason that we added MSE and coefficient of determination to all of the evaluation parts (see Table 1 and related results). We opted to maintain the findings based on correlation as they provide qualitative insights about comparative performance.**

3b) Similarly, how do you consider a median R^2 of 50% for your models as proof that flux-sums are reliable features?

**We could have used fluxes on their own as predictors of eta; however, this would have further decreased the ability to interpret the findings based on metabolites affecting fluxes by acting as substrates and/or effectors. In absence of additional means to bridge the gap between fluxes and concentrations, we relied on the concept of metabolite flux sums that has been used elsewhere (Simons *et al.* [1]). That said, future studies can inspect the effect of logistic models using fluxes of all reactions rather than pre-selected based on their contribution to metabolite flux sums.**

3c) I also maintain that your performance evaluation based on the prediction interval is not entirely meaningful. If your prediction model is bad, this evaluation will show excellent performance. And the prediction interval shown in Fig 3A spans four orders of magnitude. How is a prediction within such a range successful?

**The argument of the reviewer starts by “if your prediction model is bad, … ”. However, in our case, the Pearson correlation (for the linear model) between the predicted and estimated fluxes is 0.86 (p-value= 0.0), demonstrating a sound model based on which prediction interval is justified. This holds for all examined cases / conditions; we have included this in Supplementary Table S3.**

4) I am a bit puzzled by the distribution of the eta-values, which you provide as roughly 0.5 with not-too large standard deviation. Does that mean that—according to your method—the vast majority of reaction operate at about 50% of their maximal capacity? Is this realistic? Could you provide a plot of the probability density of eta for some scenarios?

**The distribution of calculated eta (based on known fluxes, kcat values, and enzyme abundances) is provided in the figure below. As the reviewer would notice, the eta values show a slightly right-skewed normal distribution (skewness of 0.1648), demonstrating that many reactions operate below their maximal capacities. We note that these findings assume that the entire measure abundance is used to support / drive flux.**

Minor comment:

- Fig 3A shows a substantial number of fluxes with either extremely low (zero?) estimate or prediction. Were this excluded from the evaluation and the calculation of the correlation coefficient?

**The evaluation excludes only the reactions with measured zero flux. Therefore, our findings include reactions that have predicted zero fluxes, but show measured activity.**

Reviewer #3: The authors have sufficiently addressed my previous concerns.

One remaining minor issue - the authors changed Line 27 to "that all enzymes are saturated to the same level," but this still isn't quite correct. The assumption is that each enzyme is saturated to a constant level, which may be enzyme specific but is condition invariant. Thus, v/vmax for enzyme 1 may differ from that for enzyme 2, but v/vmax does not change for enzyme 1 across conditions (a weakness of current methods for enzyme-constrained modeling). Just a minor phrasing suggestion.

**We would like to thank the reviewer for this suggestion, which we have integrated in the updated version of the manuscript.**

**References
[1] Simons M, Saha R, Amiour N, Kumar A, Guillard L, Clément G, Miquel M, Li Z, Mouille G, Lea PJ, Hirel B. Assessing the metabolic impact of nitrogen availability using a compartmentalized maize leaf genome-scale model. Plant Physiology. 2014 Nov 1;166(3):1659-74.**
